# Semantic Point Cloud Segmentation Using Fast Deep Neural Network and DCRF †

**DOI:** 10.3390/s21082731

**Published:** 2021-04-13

**Authors:** Yunbo Rao, Menghan Zhang, Zhanglin Cheng, Junmin Xue, Jiansu Pu, Zairong Wang

**Affiliations:** 1 School of Information and Software Engineering, University of Electronic Science and Technology of China, Chengdu 610054, China; m15838770021@163.com (M.Z.); xjunmin@wo.cn (J.X.); jiansu.pu@uestc.edu.cn (J.P.); 2 Yangtze Delta Region Institute (Huzhou), University of Electronic Science and Technology of China, Huzhou 313001, China; 3 Shenzhen Institutes of Advanced Technology, Chinese Academy of Sciences, Shenzhen 518055, China; zl.cheng@siat.ac.cn; 4 School of Computer Science, Neijiang Normal University, Neijiang 641100, China; wangzr@njtc.edu.cn

**Keywords:** deep learning, 3D point cloud, deep neural network, semantic segmentation, DenseCRF

## Abstract

Accurate segmentation of entity categories is the critical step for 3D scene understanding. This paper presents a fast deep neural network model with Dense Conditional Random Field (DCRF) as a post-processing method, which can perform accurate semantic segmentation for 3D point cloud scene. On this basis, a compact but flexible framework is introduced for performing segmentation to the semantics of point clouds concurrently, contribute to more precise segmentation. Moreover, based on semantics labels, a novel DCRF model is elaborated to refine the result of segmentation. Besides, without any sacrifice to accuracy, we apply optimization to the original data of the point cloud, allowing the network to handle fewer data. In the experiment, our proposed method is conducted comprehensively through four evaluation indicators, proving the superiority of our method.

## 1. Introduction

In recent autonomous driving and augmented reality applications, sensors that can directly capture 3D data are becoming more common. Extensive learning using 3D data has been extensively studied, and significant progress has been made in typical applications such as scene understanding, indoor segmentation, urban features, natural environments, shape complementation, and shape matching. However, the 3D semantic segmentation of semantic annotation of points is quite challenging. First, the sparseness of point clouds in 3D space makes most spatial operators inefficient. Due to the disorder and unstructured point cloud, the relationship between points is implicit and challenging to represent. In addition, there is a dilemma between 2D networks and 3D networks: 2D networks cannot capture 3D geometric information such as normals and shapes, while 3D networks require a large amount of computation.

Thanks to the introduction of the PointNet [1] network, we can design deep networks using point cloud data directly and end-to-end, and handle the disorder and unstructured between point clouds. Recently, Qi et al. [2,3] proposed an efficient and robust deep architecture to handle point clouds directly, which opens up new opportunities for 3D scene segmentation and motivates us to use the 3D point cloud to deal with the task of semantic segmentation.

In addition, to reduce the parameters of learning, we reduce the number of points in the scene to improve the efficiency of learning. The main contributions of our method include:

1. A point cloud reduction and feature extraction method that allows a large-scale reduction of the number of point clouds in a scene and generation of a series of ordered features of the point cloud.
2. Improved PointNet network structure is used to allow the new network structure to perform the task, and introduced a new loss function to improve the accuracy of the network.
3. A new DenseCRF functional model is proposed to make full use of the semantic classification result model to optimize the network segmentation results.

A large number of experiments were performed on different benchmark data sets to verify the proposed method and its main components. This method has achieved good results in semantic segmentation. The rest of the paper is organized as follows. Section 2 briefly reviews the related work. Section 3 describes the proposed method. Experiments and results are given and discussed in Section 4. The fifth part is a summary of the full text.

## 2. Related Work

In recent years, there has been a lot of research work on deep learning of 3D data—this section focuses on understanding the scene, such as semantic segmentation.

### 2.1. 3D Data Representation

Qi et al. [4] proposed efforts to exploit the powerful capabilities of 2D neural networks in 3D recognition. In these efforts, to adapt the input to a 2D network, a 3D scene needs to be projected into a two-dimensional plane. The 3D understanding of the scene is achieved by combining two-dimensional images from different perspectives. However, the result of this projection is the loss of many discriminating geometric details. For example, the normal vector of the point and the spatial distance are not preserved, and the front-to-back occlusion after projection hinders the overall understanding of the scene structure. These geometric details are lost, significantly limiting the accuracy of scene segmentation.

A portion of the network [5,6] focuses on using the Laplace transform to process the mesh. In addition, functional mapping [7] and loop consistency [8] help to establish the correspondence between shapes. However, this method is limited to manifold meshes. In addition, the use of mesh data requires consideration of the connection between the shape of the patch and the patch. Different choices will lead to different results.

The first attempt to apply deep learning is through volume representation. The work in [9,10,11] proposed to apply the end-to-end depth learning algorithm to 3D data analysis, including 3D shape recognition, 3D urban scene segmentation [11]. The work in [9,10] converted the original point cloud data into a voxelized occupied grid and then applied a 3D deep convolutional neural network. Nevertheless, the main challenge of volume representation is the computational overhead of data sparsity and 3D convolution. Due to the memory limitations of 3D convolution, the input voxel resolution of these methods is limited to $60^{3}$, while the depth of CNNs is also shallow, which is far from enough to represent complex shapes or scenes faithfully. To reduce the computational strength, Engelcke et al. [12] proposed to calculate the convolution of the sparse input position by pushing the value to the target position. Li et al. [13] attempts to reduce the amount of computation by sparsely sampling 3D data before entering it into the network. Tchapmi et al. [14] used a higher resolution ($100^{3}$) 3D voxel input and built a deeper network by using early downsampling and efficient convolutional blocks such as residual modules. Unlike volumetric-based or octree-based CNN methods representing a 3D shape with voxels in the same resolution, the work in [15] proposed an Adaptive Octree-based Convolutional Neural Network (Adaptive O-CNN) for efficient 3D shape encoding and decoding. Moreover, recent work [16] proposed techniques to solve the sparsity problem (for example, octree data structures). However, the performance of the capacity method is still not comparable to the point cloud-based approach.

Compared to a volume, a point cloud is a compact but intuitive representation that directly stores the geometric properties of a 3D scene through the coordinates and normals of the vertices. In the groundbreaking work of PointNet [1], the authors designed a network using unordered and unstructured point clouds. The key idea is to process the points independently and then aggregate them into a global representation through the largest pool. In the following work, PointNet++ [2], the author improved PointNet by incorporating local dependencies and hierarchical features in the network. Semantic segmentation can then be extended to graph convolution to handle large-scale point clouds [17] and KD-tree to handle non-uniform point distribution [18,19]. Meanwhile, the registration of point clouds and the detection algorithm of extreme feature areas are also constantly reducing the impact of point cloud noise [20].

### 2.2. Semantic Segmentation

There are quite a few related works on semantic segmentation. In the 3D domain, interactive semantic segmentation relies on user strokes to propagate segmentation [21,22]. For 3D segmentation, Xin Wen et al. [23] proposed new attention mechanism to predict the semantic labels. PointNet [1] and subsequent work [24,25] use multi-layer perceptron (MLP) to generate fine-grained point-level segmentation. Recently, Landrieu et al. [26,27] introduced a superpoint graph (SPG) to segment large point clouds.

Not only that, but the semantic segmentation network derived from the 3D point cloud is also becoming more and more comprehensive, and more and more attributes are used. For example, the work in [28,29], the point cloud and space where it is located are put into a graph, and the interactive relationship between the points is used to obtain a more accurate semantic segmentation result. The work in [30] goes a step further, drawing a new attention structure from the graph structure, which increases the accuracy of the semantic segmentation results.

From indoor to outdoor, the semantic segmentation scenes of point clouds are getting larger and larger, and the data volume of single scenes is also increasing, and noise problems are beginning to appear. The work in [31] proposed a new end-to-end point cloud processing network, using an adaptive sampling module to reduce the impact of noise. For the problem of fast segmentation of point cloud semantics in large scenes, the work in [32,33] proposed different complex point selection methods and local feature aggregation modules, respectively.

### 2.3. DenseCRF

Context clues represent different relationships between category labels and play an essential role in structured prediction tasks. Context semantic or higher-level information is the key to point cloud semantic segmentation. In recent years, conditional random field (CRF) is an effective method to optimize the semantic segmentation results of point clouds [34]. Combining the 3D deep network model’s feature extraction capabilities with the structured modeling capabilities of CRF can help improve the performance of point cloud semantic segmentation tasks. In general, CRFs utilize unary and binary potentials to capture the characteristics of a single 3D point and its co-occurrence [30]. In order to use the prior knowledge to enhance CRFs, high-order potentials were introduced as additional clues to aid the reasoning of semantic class labels in CRFs [35].

## 3. New Network Structure for Semantic Point Cloud Segmentation

**Figure 1:**
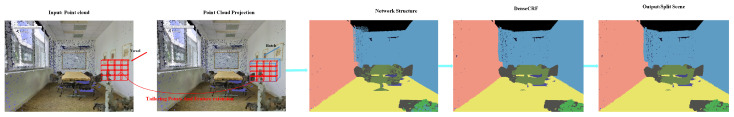
The overview of the proposed framework for semantic point cloud segmentation [36].

In this section, we first illustrate the method of point cloud mapping and feature extraction, and then introduce semantic segmentation. Given a 3D point cloud, we first map the point cloud into critical points through voxelization, and then extract more interdependent effective feature preparing for importing it into a network for further feature extraction, after which the mapped point cloud scene is imported to the network to perform semantic segmentation. To finish this task, and predict the semantic label of each point cloud of the scene, a new network structure based on PointNet is designed in our work. In the end, those labels will be merged into the DCRF model to optimize semantic segmentation. Our network structure is illustrated as shown in Figure 1, and details are described in the following sections.

### 3.1. Point Cloud Mapping and Feature Extension

**Figure 2:**
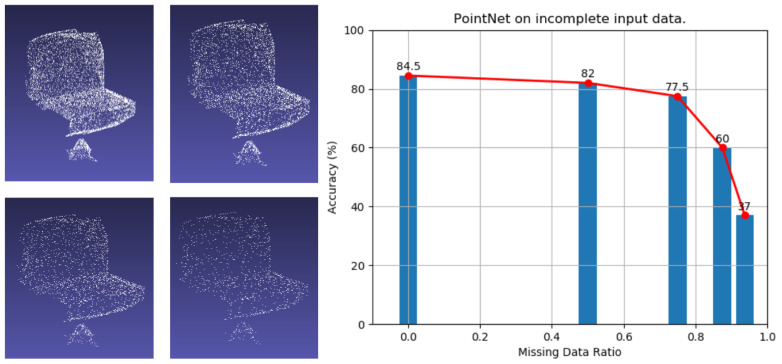
On the left is the default rate display of the point cloud. The line chart on the right describes the default recognition rate of the point cloud in the left scene, respectively.

Through the experiment, it is obvious that a non-linear relationship exists between the overall performance of the network and the number of point clouds. In detail, the number of critical points contributes to the accuracy of 3D point cloud network, shown as Figure 2. On the left of Figure 2, the point cloud’s default rate is 0%, 50%, 75% and 85%. The line chart on the right describes the point cloud’s default recognition rate in the scene as 0%, 50%, 75%, 85%, and 95%, respectively. Due to the 95% default rate of the point cloud scene can no longer be displayed normally, we do not show the 95% default rate of the point cloud scene. When continually decreasing the number of points inside a 3D point cloud, only the situations that the number of critical points is sharply cut off will cause the deterioration of the segmentation performance.

**Figure 3:**
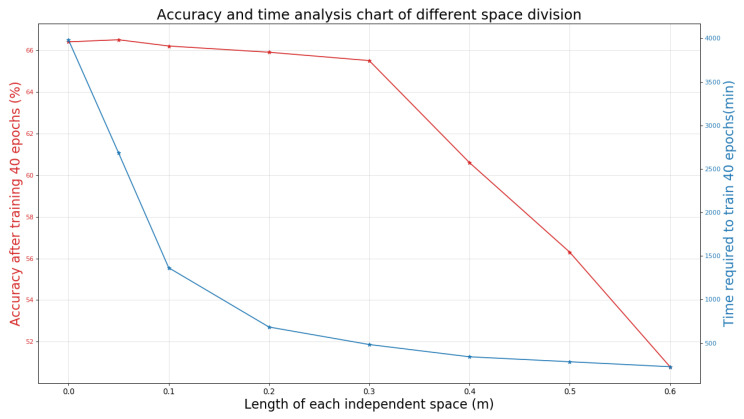
Accuracy and time analysis chart of different space division based on S3DIS dataset.

In the beginning, the 3D point cloud is divided into voxel space, in which the range of 3D coordinates is represented by the coordinates of two point clouds furthest to each other. Figure 3 depicts the comparison of the final time and accuracy when the individual spatial side length is different multiple times. The length of each voxel space is set to 0.3 m, and the defective parts are automatically filled with blanks. Here, 0.3 m is the practical value of our space division. Meanwhile, there are different experience values for the independent space division of different places, which need to be adjusted as the place changes.

It can be seen in the first half of Figure 3 that if we directly import the generated attributes of the original point cloud for training without segmentation, the result is not even better than the accuracy after processing. In our work, due to accuracy requirements, the point cloud with the correct label is divided by the total number of point clouds after all processing. Meanwhile, due to the clustering effect of point clouds and the difficulty of object boundary processing, we sparse the point clouds. The distance between the objects becomes relatively large, and the features are relatively more apparent. The accuracy of our method will be relatively improved. However, in a too sparse scene, the amount of data missing is large, and the accuracy will be significantly reduced. Also, there are many parameters involved in the election. It can be said that the size of the scene range and the sparseness of the point cloud in the scene will affect the effect of the election. However, generally speaking, the point cloud extraction situation in any scene generally conforms to the normal distribution, so we need to select the most suitable position of the confidence interval as our election point. However, the election can be improved through continuous experimentation.

**Figure 4:**
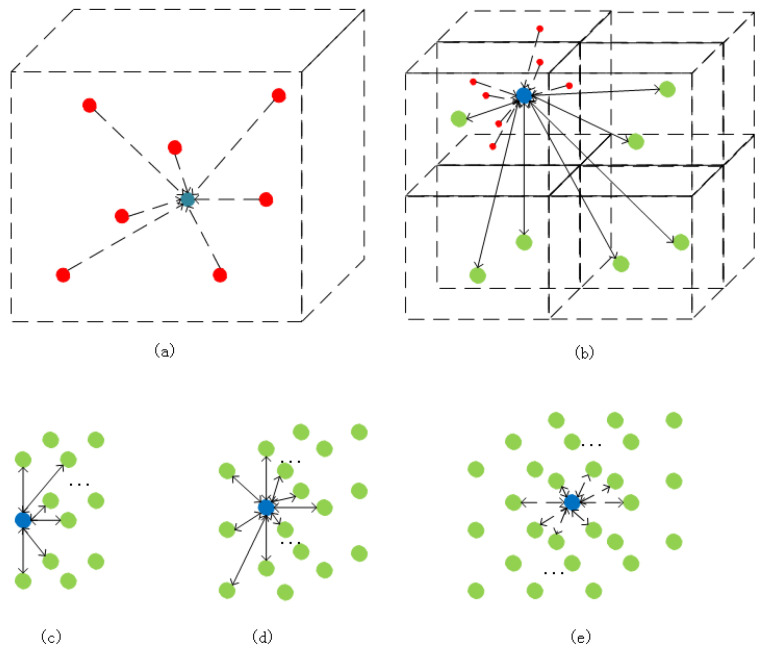
The mapped points rule of the proposed method for the neighborhood of each central point. (**a**) describes the appearance of point clouds in a divided 3D space. (**b**,**e**) describe the area that affects a single divided 3D space in the entire point cloud space. (**c**) is the position described on the edge of the entire space. (**d**) is the position described on the surface of the entire space. (**e**) is the position described on the internal area of the entire space.

Within this step, what should be paid attention to is that all point clouds within a voxel block will be aggregated for unified processing, aiming to cut off the number of points sharply. After the processing procedure, the averaged attributes of location and color will then be used as the new point cloud’s property. Furthermore, this point’s label attribute is determined by the maximum number of labels of the same label point in the space where the point is located. In order to ensure that enough points can be provided for feature extension, we establish a rule that the neighborhood of each central point must contain at least 26 mapped points; the least number of mapped point clouds that the neighborhood must enclose is 17 and 7 for point clouds on the edge and top respectively, as shown in Figure 4. Figure 4a describes the appearance of point clouds in a divided 3D space. In a single 3D space, its position information is determined by the average value of all point clouds in its space. Its color information is also determined by the average value of all point cloud color information in its space (in this single space, red represents the actual point, the blue point represents the point represented by the redpoint). (b,e) describe the area that affects a single divided 3D space in the entire point cloud space. Figure 4b describes that if the area exists at the vertex position, the area that affects its attribute extraction is the surrounding seven 3D spaces. Similarly, the position described in (c) is on the edge of the entire space, the position described in (d) is on the surface of the entire space, and the position described in (e) is in the internal area of the entire space.

Compared with the original input point cloud, the size of the election points is only one-tenth of the original points, which greatly releases the high cost of computing the network required, shown in Table 1.

In the incipient network, it utilizes only the information of coordinate, ignoring the remaining details. Other than that basic information (coordinate and RGB information), we extend a brand-new series of attributes for describing 3D point cloud based on its geometric characteristic, including scattering (Sc), linearity (Li), planarity (Pl) and verticality (Ve).

For each neighborhood, the eigenvalues of the covariance matrix of neighborhood locations are calculated as $\lambda_{1}$, $\lambda_{2}$ and $\lambda_{3}$, where $\lambda_{1}\geq \lambda_{2}\geq \lambda_{3}$. According to the optimal neighborhood principle, a proper size of the neighborhood is selected to minimize $E=-\sum_{i=1}^{3}\frac{\lambda_{1}}{\Lambda }log\left(\frac{\lambda_{1}}{\Lambda }\right)$, which is the intrinsic entropy for the vectors $\frac{\lambda_{1}}{\Lambda }$, $\frac{\lambda_{2}}{\Lambda }$, $\frac{\lambda_{3}}{\Lambda }$ and $\Lambda =\sum_{3}^{i=1}\lambda_{i}$.

(1) $$\begin{array}{c} Li=\frac{\lambda_{1}-\lambda_{2}}{\lambda_{1}},Pl=\frac{\lambda_{2}-\lambda_{3}}{\lambda_{1}},Sc=\frac{\lambda_{3}}{\lambda_{1}} \end{array}$$

Linearity reflects the extent of neighborhood growth while planarity represents if the neighborhood can be fitted by a plane, and best for loose categories like indoor plants, scattering corresponds to the disorder degree of the point clouds within its spherical neighborhood. These three features combine and form a so-called dimension.

A new attribute called verticality is introduced here, which is critical for distinguishing between planes and elevation. This attribute derives from previously defined eigenvectors $\lambda_{1}$, $\lambda_{2}$, $\lambda_{3}$ and their eigenvalue. Given that $u_{1}$, $u_{2}$, $u_{3}$ are three eigenvectors related to $\lambda_{1}$, $\lambda_{2}$, $\lambda_{3}$, the verticality is calculated as in Equation (2).

(2) $$\begin{array}{c} Ve={\left[\hat{u}\right]}_{i}\propto \sum_{3}^{j=1}\lambda_{i}\left|{\left[u_{j}\right]}_{i}\right|i=1,2,3∥\hat{u}∥=1 \end{array}$$

Defined as the weighted sum of the absolute value of the eigenvector coordinates and its eigenvalues, the vertical component of the unary vector in the main direction of 3D space represents the verticality of the point neighborhood.

### 3.2. New Neural Network for Semantics Segmentation

The network design of 3D point cloud is determined by its two diverse features: (1) It is not sensitive to the order of points, which does not affect the collection, since 3D point cloud itself is a collection of disordered points; (2) The rotation of 3D point cloud should not change the result of classification, which makes it necessary to transform the 3D point cloud data.

**Figure 5:**
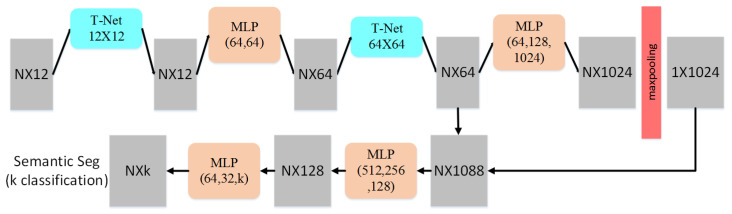
The network structure of our method [36].

As shown in Figure 5, based on PointNet structure, we construct a network for segmentation of the 3D point cloud. Given n points with 12 dimensions as input, the geometric transformation is performed with a 12 × 12 transformation network. Through MLP, each transformed point is mapped into space with a dimension of 64, in which we continue to convert them into a normalized 64-dimensional space performing high-dimensional space transformation. By applying MLP for mapping 64 dimensions to 1024 dimensions, a global feature is generated by symmetric function within the 1024-dimensional space. Finally, the classification of each point is obtained.

In our work, the loss function is inspired by [1,37,38]. Lost function in the network is calculated by summing the lost value of two parts.

(3) $$\begin{array}{c} L=L_{pred}+L_{emb} \end{array}$$

where $L_{pred}$ is the conventional cross entropy function applied in PointNet. Referring to the lost function in ASIS [37].

The $L_{pred}$ function is a regular cross entropy function that is used directly from the PointNet network. The $L_{emb}$ function is a conventional cross entropy function, which is inspired by the ASIS [37] network. The loss function $L_{emb}$ is formulated as follows:

(4) $$\begin{array}{c} L_{emb}=L_{var}+L_{dist}+\alpha L_{reg} \end{array}$$

Moreover, α is set to 0.001 in our experiment. In detail, the specific formulation of each item is as follows:

(5) $$\begin{array}{c} L_{var}=\frac{1}{K}\sum_{k=1}^{K}\frac{1}{N_{k}}\sum_{j=1}^{N_{k}}{\left[∥\mu_{k}-e_{j}∥-\delta_{v}\right]}_{+}^{2} \end{array}$$

(6) $$\begin{array}{c} L_{dist}=\frac{1}{K(K-1)}\sum_{k=1}^{K}\sum_{m=1,m\neq k}^{K}{\left[2\delta_{d}-∥\mu_{k}-\mu_{m}∥\right]}_{+}^{2} \end{array}$$

(7) $$\begin{array}{c} L_{reg}=\frac{1}{K}\sum_{k=1}^{K}{∥\mu_{k}∥}_{2} \end{array}$$

### 3.3. Improving Segmentation with DenseCRF

Assumed that V = $\left\{\begin{array}{cccc} v_{1}, & v_{2}, & \ldots , & v_{N} \end{array}\right\}$ are three dimensional point cloud for three dimensional scene, each vertex $v_{j}$ of the three dimensional point cloud is determined by its location $l_{j}$ = [$l_{j,X}$, $l_{j,Y}$, $l_{j,Z}$], color $c_{i}$ = [$c_{j,R}$, $c_{j,G}$, $c_{j,B}$], and four dimensions $d_{j}$ = [$d_{j,S}$, $d_{j,L}$, $d_{j,P}$, $d_{j,V}$], including scattering, linearity, planarity and vertivality. Letting $l^{S}=\left\{l_{1}^{S},l_{2}^{S},\ldots ,l_{N}^{S}\right\}$ be a group of semantic labels allocated to three dimensional point cloud V. We treat each vertex $v_{j}\in V$ as a node within a graph, link every two arbitrary node $v_{j}$, $v_{k}$ with an undirected edge, and associates each vertex with its semantic label $v_{j}$.

The conditional random field model is a graph model composed of unary potential and adjacent pixels, which ignores the information in the entire space. In this paper, we directly change the neighboring pixel to a point cloud, and the segmentation result obtained from the deep network is used as the input of the DCRF model. DCRF can not only use the relationship between adjacent point clouds, but also can grasp and use the pixel information of the entire space to judge and predict local point clouds. At the same time, a model can be established based on the relationship between point clouds in the space to grasp the context of the entire space fully, and its energy function is defined as:

(8) $$\begin{array}{c} E\left(l_{j}^{S}|V\right)=\sum_{j}\Phi \left(l_{j}^{S}\right)+\sum_{(j,k),j<k}\Phi \left(l_{j}^{S},l_{k}^{S}\right) \end{array}$$

We will elaborate the right three parts of the equation in the following three sections, respectively.

$\Phi (l_{j}^{S})$ denotes unary potential function. To explain it further, assumed that the semantic label set I = $\left\{\begin{array}{c} i_{1},i_{2},\ldots ,i_{k} \end{array}\right\}$ contains K semantic, and each vertex of V is distributed to these K semantic according to the current configuration of $l^{S}$, then for each semantic label i ∈ I, $\Phi (l_{j}^{S})$ can be defined as:

(9) $$\begin{array}{c} \Phi \left(l_{j}^{S}\right)=-\frac{e^{\left[-\frac{1}{2}{\left(e_{j}-\mu_{i}\right)}^{T}\sum_{i}^{-1}\left(e_{j}-\mu_{i}\right)\right]}}{\sqrt{{\left(2\pi \right)}^{d}\left|\sum i\right|}}-log\left[\sum_{k}1\left(l_{j}^{S}=i\right)\right] \end{array}$$

where $\mu_{i}$ denotes mean matrix, $\sum_{i}\left(\cdot \right)$ denotes covariance matrix, which represents the embedded item assigned to label i, 1(·) denotes an indicator that indicates whether the equation holds. In detail, 1(·) being equal to 1 means the equation holds while being equal to 0 represents the opposite.$\sum_{k}1\left(l_{j}^{S}=i\right)$ in the above formula is used for describing the range of semantics i, which is suitable to represent large-scale semantic. This item can help eliminate semantic of fine noise in point cloud.

Pairwise potential $\Phi (l_{j}^{S},l_{k}^{S})$ acquires the geometric characteristics of surface in semantic, which is defined as the gaussian mixture of the location, color, and scale information of vertex $v_{j}$ and $v_{k}$.

(10) $$\begin{array}{c} \Phi \left(l_{j}^{S},l_{k}^{S}\right)=w_{i,j}e^{\left(-\frac{{∥l_{j}-l_{k}∥}^{2}}{\lambda_{1}^{2}}-\frac{{∥c_{j}-c_{k}∥}^{2}}{\lambda_{2}^{2}}-\frac{{∥d_{j}-d_{k}∥}^{2}}{\lambda_{3}^{2}}\right)} \end{array}$$

For the formula defining $\Phi (l_{j}^{S},l_{k}^{S})$, the eigenvalues $\lambda_{1}$, $\lambda_{2}$, $\lambda_{3}$ are acquired by the characteristic matrix for corresponding location, color and dimension of vertex *j* and *k*. The definition of $w_{i,j}$ is as follows:

(11) $$\begin{array}{c} w_{i,j}=\left\{\begin{array}{cc} -1 & ifl_{j}^{S}==l_{k}^{S}\\ 1 & otherwise \end{array}\right. \end{array}$$

Therefore, the overall CRF function optimizes the scene required to be segmented, continuously adjusts the semantic label to guarantee the minimization of overall function entropy value, and acquires the best result for segmentation.

## 4. Evaluation and Comparison

### 4.1. Network Establishment and Preprocessing

We have established a novel 3D point cloud segmentation network consisting of four multi-layer perceptrons (outputs are 64, 64, 128, and 1024, respectively) and two small regularized networks. After max-pooling and the previous intermediate data, the aggregation operation is performed and divided, and the k-type semantic segmentation is to output the result. After training, the entire network model will be stored as an HDF5 format file.

Before the network is constructed, the data is preprocessed to simplify the entire point cloud’s data and extract more efficient attributes (scattering, linearity, planarity and verticality). After the end of the network, conditional random field optimization is performed on the network output semantic segmentation, and the final prediction model is output.

### 4.2. Implementation Details

**Figure 6:**
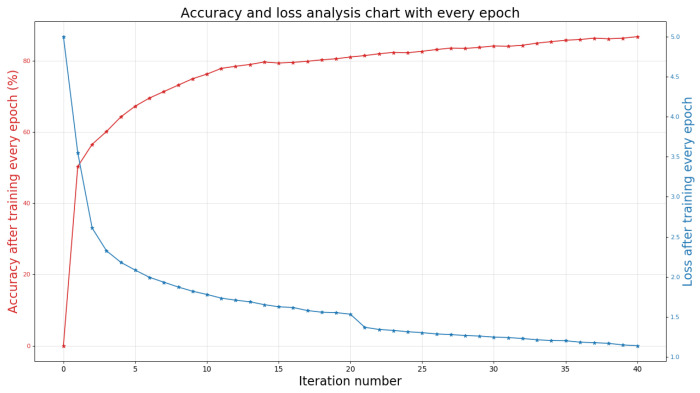
The accuracy and loss value of S3DIS data set with different iteration number [36].

After the data preprocessing and training process is completed, we input the test data set into the trained network model. After the optimization of the 3D DCRF model, the final accurate result of the 3D semantic segmentation of the scene will be generated. All work is implemented in PyTorch and runs on a server with Nvidia GeForce 1070 GPU. During the experiment, the batch size used for training was set to 1024, and the network was trained for 40 epochs. Specifically, Figure 6 shows the training accuracy and loss of the S3DIS data set every iteration. It can be seen that as the number of iterations increases, the loss rate of the model gradually decreases and tends to be stable.

### 4.3. Qualitative Evalution and Comparison

In this section, we will evaluate our proposed method on S3DIS [38] and compare the existing methods for semantic segmentation. To judge our results more objectively, we evaluate from the values of IoU, precision, recall and accuracy. Among them, these values are divided into o-prefix results for each point and m-preceding results for 13 types of average results. Next, we will give a brief introduction to each of these four criteria. Before we introduce our evaluation criteria and the sample examples in the evaluation criteria. According to traditional rules, we use TP (true positive, correctly classified positive examples), FP (false negative, originally positive examples, wrongly classified as negative examples), TN (true negative, correctly classified negative examples), and FN (False positive, originally a negative example, was divided into collation by mistake), comprehensive coverage of the classification criteria.

IOU is the ratio of the intersection and union of the two sets of true and predicted values. This ratio can be transformed into TP (intersection) than the sum of TP, FP, and FN (union):

(12) $$\begin{array}{c} IOU=\frac{TP}{FP+FN+TP} \end{array}$$

The precision rate is “really belong to category P/find belongs to category P”, and can be represented as:

(13) $$\begin{array}{c} precision=\frac{TP}{TP+FP} \end{array}$$

Recall means that for all positive examples (TP + FN) in the data set, the positive examples (TP) correctly determined by the model account for the proportion of all positive examples in the data set and can be understood as:

(14) $$\begin{array}{c} recall=\frac{TP}{TP+FN} \end{array}$$

And finally accuracy refers to the proportion of the data that the model judges correctly (TP + TN) in the total data: = (TP + TN)/(TP + FP + TN + FN).

(15) $$\begin{array}{c} accuracy=\frac{TP+TN}{TP+FP+TN+FN} \end{array}$$

**Figure 7:**
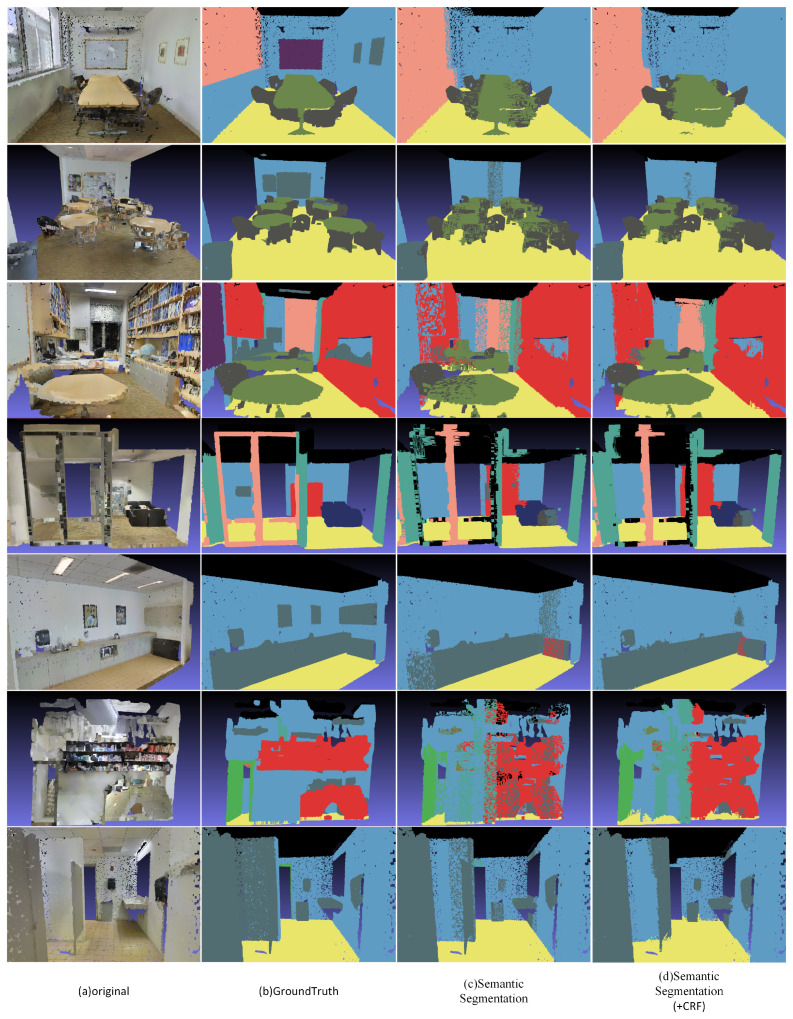
The semantic segmentation results of the proposed method based on S3DIS data set [36].

The results of our method are shown in Figure 7. The first column represents the original 3D point cloud scene; the second column represents the ground truth of the scene that appeared in the first column; the third column represents the results processing the original scene without optimizing through CRF; the fourth column represents the segmentation result with CRF optimization. It can be seen from the segmentation results that our method can fuse global point cloud information with a single point cloud information by using a special and effective 3D neural network architecture and combine 3D DCRF to optimize the semantic segmentation boundary, thereby accurately segmenting different semantic objects. It is worth mentioning that our method can accurately segment different semantics in conventional and unconventional situations. However, due to the large amount of noise in the scene, we will have a large number of intersection point clouds between the two different semantics under the initial segmentation. In contrast, our method can clearly separate these intersection point clouds. Under the framework of unified multitasking, the 3D point cloud neural network and the 3D DCRF can qualitatively find and predict the filling of point cloud information, thereby enhancing its advantages.

**Figure 8:**
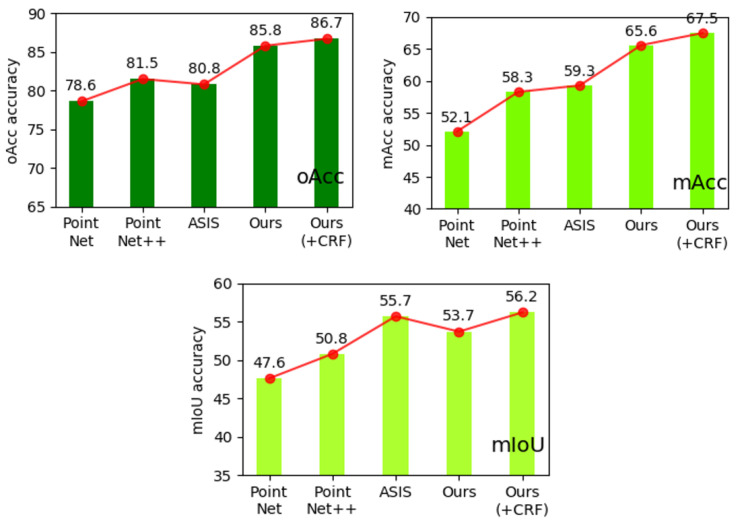
Semantic segmentation mIoU, oAcc and mRecall on S3DIS. This figure shows that our network is second on mIOU without CRF optimization and is optimal on both mACC and oAcc. After joining CRF optimization, our network will be able to achieve the best in the compared network.

Table 2 shown a comparison results of semantic segmentation based on metric:mIoU, mACC and mRecall. From Table 2, we can find our method with DCRF get value (mIoU = 65.2, mAcc = 67.5, mRecall = 0.372), which is better than the PointNet, PointNet++, and ASIS. If the proposed method does not use DCRF, its value is smaller. Figure 8 display the difference of performance on S3DIS dataset between our proposed method and three other ones based on several benchmarks, including mIoU, mACC and mRecall. Among them, PointNet [1] and PointNet++ [2] are the most important works of point cloud semantic segmentation. ASIS [37] is similar to us; both are networks that integrate the results of semantic segmentation. Our network has been greatly improved compared with [1,2]’s work, and it has made great progress for another converged network.

In addition, we also analyze the accuracy of each class of S3DIS and compare its result with three other networks, shown in Table 3. The networks we compared are PointNet [1], Pointwise [39] and SEGCloud [14]. The pointwise network is a unique research achievement in point cloud semantic segmentation, a network that performs convolution point by point and completes the final semantic segmentation result. Simultaneously, the SEGCloud network is an excellent network that introduces a new structure in the point cloud semantic segmentation. Our network and these three networks are compared in 13 semantic classes. Except for some semantic classes, our segmentation results have achieved excellent results.

Table 2 and Table 3 show that the networks compared by this network are PointNet and PointNet++, two classic networks. There are three different types of networks: PointWise networks based on point-by-point convolution and based on voxel segmentation. The SEGCloud network and the ASIS network are based on MLP. These networks basically represent all the larger categories of point cloud semantic segmentation. Since our network does not have much design in the training of using local information, when we choose the comparison network, we also choose some networks that do not use too much local information.

From the perspective of time efficiency and segmentation efficiency, we believe that our network can achieve a better balance between the segmentation effect and time efficiency under the same conditions. If you need to pursue higher segmentation efficiency, the main body of the network can be redesigned. For example, an attention mechanism can be added to the main body of the semantic segmentation network to improve the segmentation accuracy. Therefore, the process of this network is divided at the beginning of the design to facilitate subsequent network upgrades and improvements.

While this network is directly improved and applied from the mainframe network of PointNet, the use of local information in the overall point cloud space is not as good as the current multi-parameter multi-layer network structure, especially the network with the attention mechanism causes the complexity of the network significantly increased. A point cloud semantic segmentation network with an attention mechanism has 3–4 layers more than the ordinary point cloud semantic segmentation network. The number of parameters is more than doubled, which increases the difficulty and time efficiency of network training. In the case of the same design structure, our network can achieve better results with only a straightforward network structure. Under the same data set, such as the S3DIS data set, use area1 to area5 for training and area6 for testing. On NVIDA1070, the training time using the ordinary PointNet network is 23 h, the training time using PointNet++ is 33 h, and the training time using the ASIS network is 31 h, but our network only needs 8 h. In the process of increasing the number of network layers, the training time will gradually increase, but the training efficiency cannot be significantly improved.

**Figure 9:**
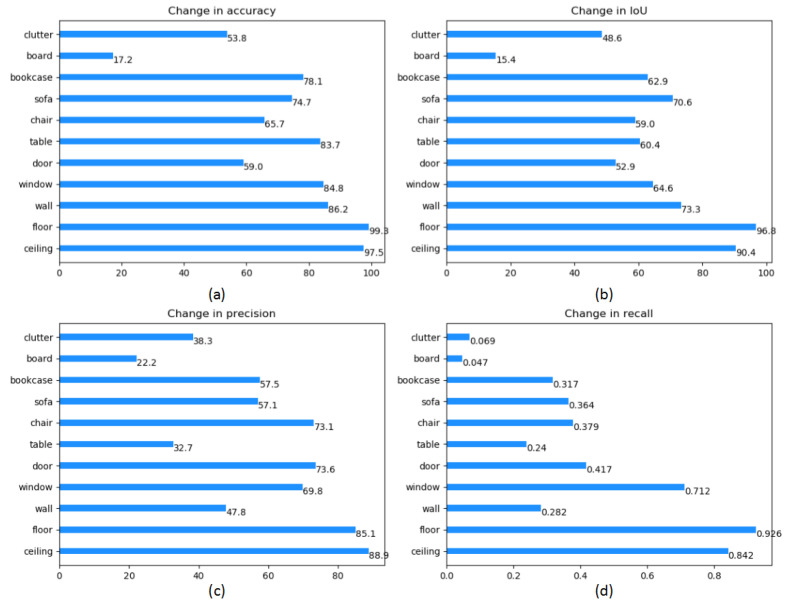
Semantic segmentation mIoU, oAcc and mRecall on S3DIS [36]. (**a**) is accuracy results of semantic segmentation. (**b**)is IoU results of semantic segmentation, (**c**) is precision results of semantic segmentation, (**d**) is recall results of semantic segmentation.

In the end, judged by four indicators, including accuracy, IoU, precision and recall, we display the result of each class in the dataset, which is shown in Table 4 and Figure 9. Compared to those methods that achieve state-of-art performance, our proposed method shows significant improvements in some categories (e.g., floor, sofa and table) while suffering from slightly lower accuracy on a few specific categories.

## 5. Conclusions

This paper proposes a novel network structure that improves semantic segmentation results. By applying semantic segmentation to produce a segmentation model, our network achieves high accuracy on semantic segmentation, taking CRF as a post-processing step. At the same time, performing mapping and feature extension to point cloud data in the stage of preprocessing, the number of network arguments is sharply cut off without decreasing the overall performance. Compared to other methods, we evaluate our proposed methods on S3DIS indoor dataset, which shows that the proposed method has good segmentation performance and can be easily generalized into other segmentation tasks within various point cloud scenes.

## Figures and Tables

**Table 1:** Comparison of point numbers before and after dataset scene point cloud mapping in S3DIS (PN = Points Number).

Scene (Area5)	Original Projection (PN)	Election Projection (PN)	Compression Ratio (%)
conferenceRoom1	1,047,554	118,784	11.34
lobby1	1,223,236	131,072	10.72
office10	752,349	90,112	11.98

**Table 2:** Semantic segmentation mIoU, oAcc and mRecall on S3DIS. (a) is accuracy results of semantic segmentation. (b) is IoU results of semantic segmentation, (c) is precision results of semantic segmentation, (d) is recall results of semantic segmentation.

Method	mIoU	mAcc	mRecall
PointNet [1]	47.6	52.1	-
PointNet++ [2]	50.8	58.3	-
ASIS [37]	55.7	59.3	-
Ours	53.7	65.6	0.338
Ours (+CRF)	56.2	67.5	0.372

**Table 3:** Evaluated the accuracy of each of our classes on the S3DIS dataset and compared it to three other networks on S3DIS.

Method	oAcc	Ceiling	Floor	Wall	Window	Door	Table	Chair	Sofa	Bookcase	Board	Clutter
PointNet [1]	78.6	88.8	97.3	69.8	46.3	10.8	52.6	58.9	40.3	5.9	26.4	33.2
Pointwise [39]	81.5	97.9	99.3	92.7	49.6	50.6	74.1	58.2	0	39.3	0	61.1
SEGCloud [14]	80.8	90.1	96.1	69.9	38.4	23.1	75.9	70.4	58.4	40.9	13	41.6
Ours	85.8	96.5	99.2	86.5	82.4	57.3	81.1	65.3	67.9	74.3	16.3	55.8
Ours(+CRF)	86.7	97.5	99.3	86.2	84.8	59.0	83.7	65.7	74.7	78.1	17.2	53.8

**Table 4:** Evaluated the change of each target of our classes on the S3DIS dataset. CA = Change in accuracy, CIoU = Change in IoU, CP = Change in precision, CR = Change in recall.

	Ceiling	Floor	Wall	Window	Door	Table	Chair	Sofa	Bookcase	Board	Clutter
CA	97.5	99.3	86.2	84.8	59.0	83.7	65.7	74.7	78.1	17.2	53.8
CIoU	90.4	96.8	73.3	64.6	52.9	60.4	59.0	70.6	62.9	15.4	48.6
CP	88.9	85.1	47.8	69.8	73.6	32.7	73.1	57.1	57.5	22.2	38.3
CR	0.842	0.926	0.282	0.712	0.417	0.240	0.379	0.364	0.317	0.047	0.069

## Data Availability

Dataset from: http://buildingparser.stanford.edu/dataset.html.
